# Harmine, an inhibitor of the type III secretion system of *Salmonella enterica* serovar Typhimurium

**DOI:** 10.3389/fcimb.2022.967149

**Published:** 2022-09-13

**Authors:** Yunjia Shi, Xindi Chen, Jingyan Shu, Yang Liu, Yong Zhang, Qianghua Lv, Jianfeng Wang, Xuming Deng, Hongtao Liu, Jiazhang Qiu

**Affiliations:** ^1^ Laboratory for Zoonotic Diseases, Ministry of Education, College of Veterinary Medicine, Jilin University, Changchun, China; ^2^ Department of Respiratory Medicine, Center for Pathogen Biology and Infectious Diseases, Key Laboratory of Organ Regeneration and Transplantation of the Ministry of Education, State Key Laboratory for Zoonotic Diseases, The First Hospital of Jilin University, Changchun, China

**Keywords:** Harmine, *S.* Typhimurium, SPI-1, type III secretion system, HilA

## Abstract

New therapeutic strategies for clinical *Salmonella enterica* serovar Typhimurium (*S.* Typhimurium) infection are urgently needed due to the generation of antibiotic-resistant bacteria. Inhibition of bacterial virulence has been increasingly regarded as a potential and innovative strategy for the development of anti-infection drugs. *Salmonella* pathogenicity island (SPI)-encoded type III secretion system (T3SS) represents a key virulence factor in *S.* Typhimurium, and active invasion and replication in host cells is facilitated by the secretion of T3SS effector proteins. In this study, we found that harmine could inhibit T3SS secretion; thus, its potential anti-*S*. Typhimurium infection activity was elucidated. Harmine inhibits the secretion and expression of T3SS effector proteins and consequently attenuates the *S.* Typhimurium invasion function of HeLa cells. This inhibition may be implemented by reducing the transcription of pathogenesis-related SPI-1 transcriptional activator genes *hilD*, *hilC*, and *rtsA*. Harmine improves the survival rate and bacterial loads of mice infected with *S*. Typhimurium. In summary, harmine, an effective T3SS inhibitor, could be a leading compound for the development of treatments for *Salmonella* infection.

## Introduction

*Salmonella enterica* serovar Typhimurium (*S.* Typhimurium) is the most frequent foodborne bacterial pathogen, and it causes enteritis, diarrhea and life-threatening invasive diseases in a wide range of hosts (Coburn et al., 2007). It represents a continuing threat to poultry industries (Sockett, 1995; Plym and Wierup, 2006). *S.* Typhimurium has developed varying degrees of antibiotic resistance, thus rendering traditional antibiotic therapy treatment for bacterial infections ineffective (Walther et al., 2016). Therefore, it represents a serious problem, and novel anti-infective therapies are urgently required (Nesterenko et al., 2016). One promising strategy for the discovery of novel antibacterial agents is to target the functions essential for inhibiting bacterial infection, such as virulence factors. *Salmonella* type III secretion system (T3SS) is a virulence apparatus that promotes invasion and dissemination and is also a typical representative virulence factor target for the development of antivirulence therapies for innovative agents against bacterial infection (Aiello et al., 2010). Structural genes of T3SS apparatus, several effector proteins and the transcriptional regulators are encoded by *Salmonella* pathogenicity island (SPI) (Ellermeier and Slauch, 2007). The master SPI-1 regulatory gene hyperinvasion locus A (*hilA*) is directly activated by three AraC-like regulators, HilD, HilC, and RtsA, which induce the SPI-1 locus (Ellermeier and Slauch, 2003). In addition, HilD is the dominant SPI-1 regulatory circuit regulator, while HilC and RtsA amplify these inducing signals, thus creating a complex feed-forward regulatory loop (Ellermeier et al., 2005). These findings support the hypothesis that targeting the feed-forward regulatory loop may impair *Salmonella* virulence (Kim et al., 2019). The SPI-1-associated T3SS bacterial effector proteins SipA, SipB, SipC and SopB were internalized into the host cell cytosol (Moest and Méresse, 2013) and are essential for *Salmonella* virulence and invasion because they destroy the immune defense by inducing host cell membrane ruffles (Diepold and Armitage, 2015). Thus, T3SS is a new drug target for the discovery of antibacterial agents.

Harmine is isolated from seeds of *Peganum harmala* and possesses anxiolytic and behavioral effects (Wu et al., 2020). It exhibits various bioactivities, and recent studies confirmed the antiviral activities of harmine against dengue virus (Quintana et al., 2016), herpes simplex virus type 1 and type 2 (Benzekri et al., 2017), and enterovirus 71 (Chen D et al., 2015). Harmine can inhibit the growth of several types of cancer cells based on its antitumor effects (Hamsa and Kuttan, 2011) and reverse resistance to anticancer drugs (Ma and Wink, 2010). Harmine has also shown remarkable inhibitory activity against *Fusarium moniliforme*, but the mechanism has not been clarified (Zaidi et al., 2004). The anti-inflammatory activity of harmine was demonstrated through its strong ability to suppress tumor necrosis factor-alpha and nitric oxide production in RAW264.7 and human THP-1 cells (Filali et al., 2015). In addition, the neuroprotective effects of harmine could be ascribed to its ability to reduce oxidative stress (Réus et al., 2010) and inflammation (Riquelme et al., 2016). Although harmine has many pharmacological activities in previous reports, the effect of harmine on *S*. Typhimurium infection and *S*. Typhimurium T3SS function has not been reported.

In this study, harmine was first proved to be an T3SS inhibitor of *S*. Typhimurium. The therapeutic efficacy of harmine on *S*. Typhimurium infection was evaluated in a mouse model. Together, the data suggested that harmine could serve as a potential novel antibacterial agent for *Salmonella* infection and provided evidence-based data for clinical application.

## Materials and methods

### Bacterial strains and culture conditions

The *S.* Typhimurium strain SL1344 and its T3SS-defective mutant strain (SL1344 Δ*invA* variant) used in this study were provided by Dr. Xiaoyun Liu from Peking University. SL1344 chromosomally expressing Flag-tagged SipB (SipB-3×Flag) were cultured in LB broth (30 μg/mL streptomycin). SL1344 carrying the vector of SipA-beta-lactamase (SipA-TEM) was grown aerobically in Luria-Bertani (LB) broth (100 μg/mL ampicillin) at 37°C. These strains were preserved in our laboratory.

### Effect of harmine on the translocation of effector SipA-TEM

The SipA-TEM fusion reporter system and flow cytometry were used to investigate the transfer of the effector SipA into eukaryotic host cells. For SipA-TEM assay, HeLa cells (1.2×10^4^ cells/mL) were cultured in 96-well plates. Overnight cultures of SL1344 or SL1344 Δ*invA* expressing SipA-TEM was diluted 30-fold in fresh 0.3 M NaCl medium cultures. A gradient concentration (0, 4, 8, 16, 32 μg/mL) of harmine (purity=99.826%, Herb-purify, Chengdu, China) was added, and the cells were cultured for another 4 h. In the control group, dimethyl sulfoxide (DMSO; Sigma-Aldrich) was added in the same volume. Bacterial cultures were centrifuged, the supernatant was discarded, and the pellet was resuspended with PBS. Then, the cells were infected with bacteria (MOI=20) and incubated at 37°C for 1 h. The TEM substrate 6×CCF4/AM reagent (K1095, Life Technologies) was added to each well for 1 h at ambient temperature and visualized using fluorescence microscopy (IX83, Olympus).

For flow cytometry, HeLa cells seeded in 24-well plates were infected and allowed to react with CCF4/AM as described above. The cells were washed with PBS and collected by trypsinization and centrifuged for 5 min at 1000 rpm. Pelleted cells were gently resuspended in 400 μl PBS and analyzed on a flow cytometer to quantify the blue fluorescence.

### Effect of harmine on the expression and secretion of endogenous SPI-1 effector proteins

An SPI-1-associated effector protein secretion assay was described as previous reports (Negrea et al., 2007). The overnight cultures of SL1344 were inoculated in LB broth. The overnight culture was diluted 1:30 and grown in LB broth (0.3 M NaCl; 30 μg/mL streptomycin) with or without harmine at a linear concentration-dependent increase and grown for 4 h at 37°C. Secreted proteins in 1.5 mL supernatant were treated with 10% trichloroacetic acid (TCA) precipitation. The post incubation cultures were collected by centrifugation at 14,000×*g* for 20 min and washed twice with ice-chilled acetone. Then, the samples were allowed to dry for 15 min before the resulting precipitated proteins were dissolved in 4% SDS loading buffer. Visualization of the secreted *S.* Typhimurium T3SS cargo was analyzed by SDS-PAGE and stained with Coomassie blue. The total culture was visualized by WB as detailed below.

### Effect of harmine on the expression of SPI-1 gene-encoded proteins

For the western blotting (WB) experiments, SL1344 SipB-3×flag was cultured in LB at 37°C overnight. Next, the cells were diluted 1:30 into fresh LB supplemented with 0.3 M NaCl (30 μg/mL streptomycin) and grown by the addition of harmine for 4 h at 37°C. Whole bacterial cells were collected by centrifugation at 12,000×*g* for 2 min. The cell pellets were mixed with 50 μL of 1×sodium dodecyl sulfate (SDS) loading buffer, heated for 5 minutes at 95°C, separated by 10% SDS-PAGE and then blotted onto a PVDF membrane. Proteins were analyzed by western blotting with HilA rabbit anti-HilA IgG (1:500; prepared by our laboratory), SipA rabbit anti-SipA IgG (1:500; prepared by our laboratory), rabbit anti-isocitrate dehydrogenase (ICDH) IgG (1:20,000; ABS2090, Sigma), and anti-Flag mouse IgG (1:5,000; F1804, Sigma). After washing three times with PBST, the membrane was incubated with HRP-conjugated secondary antibody with goat anti-rabbit IgG H&L labeled with Alexa Fluor-790 (ab175781, Abcam) and goat anti-mouse IgG H&L labeled with Alexa Fluor-680 (ab175775, Abcam).

### Effect of harmine on the transcription of SPI-1 genes

Real-time quantitative PCR (qRT-PCR) was used for the analysis of SPI-1 gene transcription. SL1344 was cultured overnight, and the cultures were diluted 1:30 in LB broth (0.3 M NaCl; 30 μg/mL streptomycin) with harmine by gradient concentrations (0, 8, 16, 32 μg/mL) and grown at 37°C for another 4 h. Total bacterial RNA was extracted using a Bacterial Total RNA Extraction kit (B518625, Sangon Biotech), and cDNA was amplified using the RevertAid RT reverse transcription Kit (K1691, Thermo Scientific) by following the manufacturer’s instructions. The qRT-PCR were then performed using SYBR Green fluorescent dye (KTSM1401, AlpaLife). All PCR experiments were analyzed in three replicates. The primer pairs are listed in **Table 1**.

**Table 1:** Primers for RT-PCR in this study.

Gene name	Primer sequence (5’-3’)	Product size (bp)
*sipA*	CCGGCACCTTGAAATGCAAA	385
	CGAATCCACACGCGAATGAC	
*sipB*	ATGGGATGTATCGGGAAAGT	360
	CTCCATAATCGGGTTTAGCG	
*sipC*	CAGCTTCGCAATCCGTTAGC	358
	TCAGCCTGGTTCAACGTCAG	
*hilA*	TATCTCCGGGCAGATGATAC	340
	TCTGAGCAAAAGATTCGCAA	
*hilC*	AGCGTATCAAGTCTGAAGCG	147
	ATCATAGCCACACATCGTCG	
*hilD*	TAACGTGACGCTTGAAGAGG	123
	GGTACCGCCATTTTGGTTTG	
*rtsA*	AGGTGGGGAGCATTGAAT	125
	CGTAATTGAAATTTTACCC	
*gyrB*	TCATTTCCACTACGAAGGCG	111
	CCGAAAAAGACGGTATCGG	

### Effect of harmine on growth of *S*. Typhimurium

Overnight cultures of SL1344 were diluted at a ratio of 1:30 into 100 mL of fresh LB supplemented with 0.3 M NaCl in the presence of harmine by gradient concentrations (0, 2, 4, 8, 16, 32 μg/mL). OD_600_ was measured until it reached the stationary growth phase (30 min intervals). After incubation in the presence or absence of harmine for 5 h at 37°C, the OD_600_ values were measured by spectrophotometry (Biophotometer, Eppendorf) every hour for 8 h.

### Effect of harmine on the motility of *S.* Typhimurium

The addition of 1 μL of SL1344 from the overnight growth culture was spotted onto 0.3% LB agar plates containing an equivalent volume of DMSO or harmine at a gradient concentration and were stab-inoculated in six-well plates. The agar plates were cultured at 37°C overnight before the diameter (mm) of motility halos was measured. Three biological replicates were used for each sample.

### Cytotoxicity analysis of harmine in HeLa cells

Lactate dehydrogenase (LDH) reagent was used to examine the cytotoxicity of harmine. HeLa cells were placed in a 96-well culture plate and treated with 0, 2, 4, 8, 16 and 32 μg/mL harmine for 12 h. Subsequently, cell viability was assessed by measuring LDH release in culture supernatants using the Cytotoxicity Detection Kit (11644793001, Roche).

### Gentamicin protection assay and immunofluorescence analysis

The effect of harmine on bacterial invasion of mammalian cells was verified by gentamicin protection with minor modifications, as previously described (Galan and Curtiss, 1989). HeLa cells (2×10^5^; in DMEM, 10% FBS) were cultured on 24-well plates and incubated at 37°C in a CO_2_ incubator. Bacterial cultures were cultured overnight at 37°C in a shaker set and then subcultured (1:30) in fresh 0.3 M NaCl LB with different concentrations of harmine or an equal volume of DMSO for 3 h. Bacterial inoculates were diluted in sterile PBS and added to cells at a multiplicity of infection (MOI) of 20 at 37°C for 1.5 h. The culture media was replaced with fresh DMEM containing gentamicin (100 μg/mL) to kill extracellular bacteria at 37°C for another 1 h. At 2 h post-infection, saponin-permeabilized cell and CFUs were determined by appropriate dilutions. Cells were fixed onto cover slips by incubation in 4% PFA in PBS for 20 min at room temperature before permeabilization by using 0.02% (v/v) Triton X-100 in PBS for 5 min at room temperature. After blocking by incubating the cells with 4% goat serum (20 min at room temperature), coverslips were stained with anti-*S.* Typhimurium primary antibodies (ab35156, Abcam; 1 h at room temperature) and subsequently incubated with fluorescein-labeled secondary antibodies (A21206, Life Technologies) for 1 h at room temperature. The nuclei were stained with Hoechst 33342 (C1025, Beyotime), and the immunofluorescence analysis was performed with an Olympus fluorescence microscope (IX83, Olympus).

### Animal experiments

Female BALB/c mice (6-8 weeks old, 18-20 g) were purchased from Liaoning Changsheng Biotechnology Co., Ltd. and maintained on a 12-h light-dark cycle. All animal experiments were conducted based on protocols approved by Jilin University (number of permit: 2021100893G). Drinking water containing antibiotics (streptomycin, 5 g/L) was provided for three days before SL1344 infection (Barthel et al., 2003). For the survival assays, each mouse was orally inoculated of SL1344 (1×10^7^ colony-forming units (CFUs) in 100 μL of PBS) using a gavage needle and the control groups were administered the same volume of sterile PBS without bacteria (n=10 for each group). Harmine was offered 2 h before SL1344 infection at a dose of 100 mg/kg, followed by oral administration for another 1-4 days at 12-h intervals in succession. We monitored the mice for 10 days after infection. To determine the bacterial load in the spleens and livers of mice infected with SL1344 by 5×10^6^ CFUs, tissue samples were homogenized 4 days post infection for bacterial quantification in cold PBS (n=5 for each group). The number of CFUs in the organ homogenates was determined by serial dilution and plating on 30 μg/mL streptomycin agar plates, followed by overnight incubation at 37°C. All experiments were performed in triplicate.

### Statistical analysis

Statistical data are presented as the mean of at least three replicates ± standard deviation. Statistical analyses were performed using unpaired two-tailed t tests, and the log-rank test was used for mouse survival assays (GraphPad Software Inc.; La Jolla, CA, USA). Probability (*P*) values of < 0.05 were considered significant. (*P < 0.05; **P < 0.01; NS, *P* > 0.05, not significant).

## Results

### Harmine inhibits the translocation of SipA

**Figure 1:**
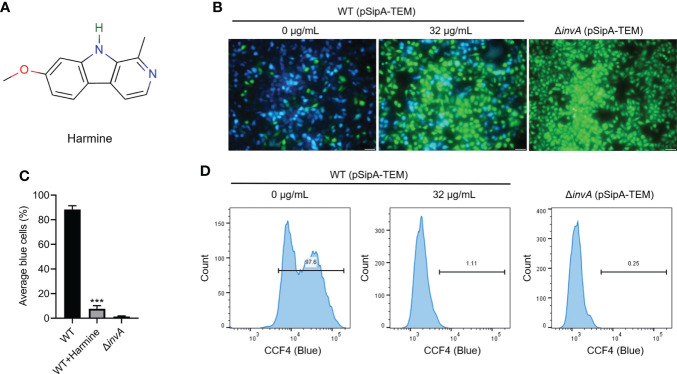
Harmine inhibits the translocation of SipA-TEM *via* the T3SS of *S.* Typhimurium. **(A)** Chemical structure of harmine. **(B)** Representative images of the effect of harmine treatment on the translocation of SipA**-**TEM (scale bar, 50 μm). Fluorescence microscopy images were acquired to analyze positive and negative protein translocation efficiency by measuring blue and green cells. **(C)** Three independent experiments were performed to test and quantitatively analyze the blue fluorescence signals of the cells. **(D)** Harmine (32 μg/mL) was used to analyze *S.* Typhimurium invasion of HeLa cells by flow cytometry. HeLa cells infected with the SipA**-**TEM strains measured using the FRET substrate CCF4/AM were analyzed by flow cytometry. The experiment was performed in triplicate. ***P < 0.001.

The SL1344 SipA-TEM reporting system was used to investigate the effect of harmine on the translocation of *S*. Typhimurium T3SS effector proteins (Yahr and Wolfgang, 2006). The results of preliminary screening assay of T3SS inhibitors from natural compounds using SipA-TEM reporter system showed that 64 μg/mL of harmine inhibited SipA-TEM fusion translocation to HeLa cells. Harmine is a tricyclic β-carboline alkaloid with a molecular formula of C_13_H_12_N_2_O and a molecular weight of 212, and its chemical structure is shown in **Figure 1A**. The results showed that harmine inhibited SipA-TEM translocation into HeLa cells at 32 μg/mL (**Figures 1B, C**). As expected, the 32 μg/mL harmine-treated group showed reduced blue (cleaved) fluorescence (1.11%) compared with the WT strain group (97.6%), as analyzed by flow cytometry (**Figure 1D**). HeLa cells infected with SL1344 Δ*invA* (0.25%) (blue) were used as the harmine-treated group (blue) (**Figure 1D**). These results illustrated that harmine inhibited the translocation of SipA.

### Harmine inhibits the secretion of T3SS effector proteins

**Figure 2:**
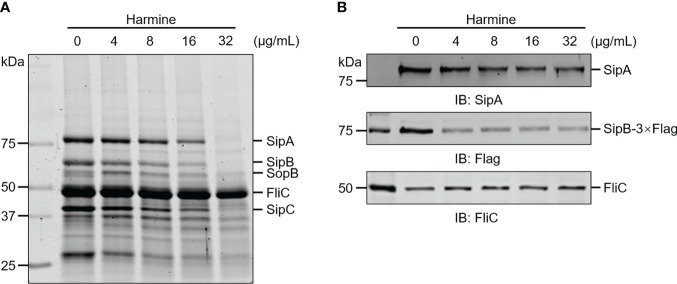
Harmine inhibits the secretion of *S*. Typhimurium SPI-1 T3SS effector proteins in a dose-dependent manner. **(A)** Coomassie blue staining of culture supernatants were assessed to assay *S.* Typhimurium T3SS effector protein secretion. **(B)** WB of culture supernatants were assessed to assay SipA and SipB secretion. FliC, flagellar filament protein.

The amount of secreted protein was reduced upon harmine treatment in a dose-dependent manner (0-32 μg/mL) and was analyzed by SDS-PAGE based on Coomassie brilliant blue staining (**Figure 2A**). WB was further used to examine these inhibitory phenotypes (**Figure 2B**). Harmine showed an inhibitory effect on the secretion of the T3SS effectors (SipA, SipB, SipC and SopB) without affecting the housekeeping protein FliC (**Figures 2A, B**). These data show that harmine inhibits the secretion of T3SS effector proteins.

### Harmine inhibits the expression of T3SS effector proteins

**Figure 3:**
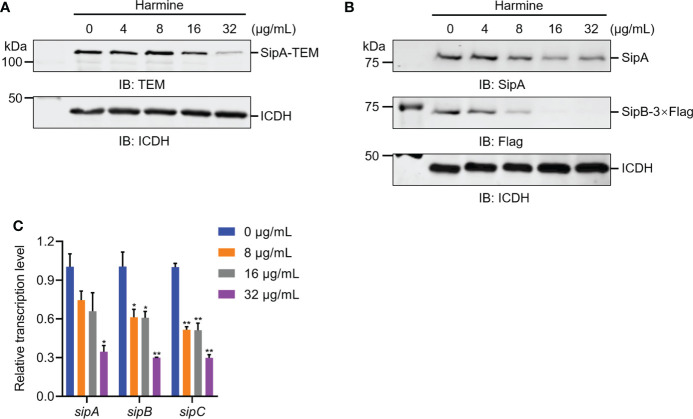
Harmine inhibits the expression of the *sipA* and *sipB* genes by reducing the transcriptional level of SPI-1 T3SS effector proteins. **(A)** Effect of harmine on SipA**-**TEM expressed in *S*. Typhimurium by WB analysis. The metabolic enzyme isocitrate dehydrogenase (ICDH) was used as a loading control. IB, immunoblotting. **(B)** Effect of harmine on the expression of the *sipA* and *sipB* genes by WB analysis. ICDH was used as a loading control. IB, immunoblotting. **(C)** Effect of harmine on the relative mRNA levels of the *sipA* and *sipB* genes by qRT-PCR. The control group was added with the same volume of dimethyl sulfoxide. The *gyrB* gene was used as the endogenous control. *P < 0.05, **P < 0.01.

To explore the possible molecular mechanism underlying harmine inhibition of *S.* Typhimurium T3SS effector secretion, we measured related effector protein expression levels *via* WB analysis and qRT-PCR analysis. As expected, the identities of the protein bands in **Figures 3A, B** showed that the harmine pretreated group protein molecular weights were significantly decreased for the SL1344 SipA-TEM and endogenous effector proteins SipA, SipB and SipC compared to the untreated group, with the changes showing a dose-dependent trend (**Figures 3A, B**). The mechanism of harmine’s action was determined by measuring the mRNA transcription levels of T3SS genes by qRT-PCR (**Figure 3C**). Consistent with the expression in the control group, the gene expression of the T3SS effector proteins was significantly downregulated in the harmine pretreatment group in a dose-dependent manner (**Figure 3C**). Compared with the mRNA levels, the trend of all tested genes was reduced, which was similar to the WB analysis (**Figures 3A, B**). Overall, harmine showed strong inhibitory activity against the mRNA transcription of the T3SS gene and therefore reduced the expression of T3SS functional proteins.

### Harmine inhibits T3SS mainly by targeting HilA

**Figure 4:**
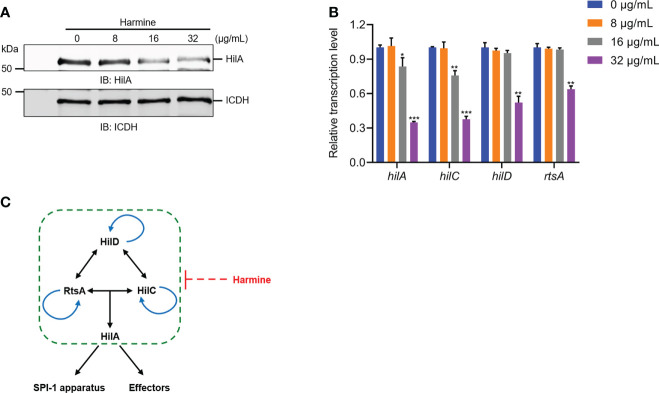
Harmine inhibits the transcription of the SPI-1 regulatory gene *hilA* by inhibiting the transcription of three AraC-like regulators. **(A)** Effect of harmine on the expression of *hilA* genes by WB analysis. ICDH was used as a loading control. IB: immunoblotting. **(B)** Effect of harmine on the transcription of the regulatory genes *hilD, hilC, rtsA* and *hilA* by qRT-PCR. **(C)** Schematic diagram of harmine on SPI-1 regulation. Arrows indicate positive effects. Blunt line ends designate negative effects. *P < 0.05, **P < 0.01, ***P < 0.001.

Guided by these results, we explored the regulatory factor proteins and investigated the expression of HilA with or without harmine at different concentrations using WB and qRT-PCR assays. Harmine caused a reduction in the expression of HilA in a dose-dependent manner, as shown by the WB analysis (**Figure 4A**); moreover, the transcription levels of *hilA*, *hilD, hilC* and *rtsA* were inhibited by harmine in a dose-dependent manner (**Figure 4B**). HilA is the critical regulator of SPI-1-induced T3SS that is known to combine with the promoters of the AraC-like regulator genes *invF* and *sicA* and directly regulates the transcriptional downstream genes, which are targets of SPI-1 apparatus genes and T3SS effectors (Golubeva et al., 2012). The transcription of *hilA* is controlled by a feed-forward regulatory loop formed by HilC, HilD, and RtsA of AraC-like proteins (Schechter and Lee, 2001). These three regulators can activate each other and HilA (**Figure 4C**). In short, harmine inhibited the transcription of SPI-1-related genes through a feed-forward regulatory loop.

### Harmine inhibited *S.* Typhimurium invasion of HeLa cells

**Figure 5:**
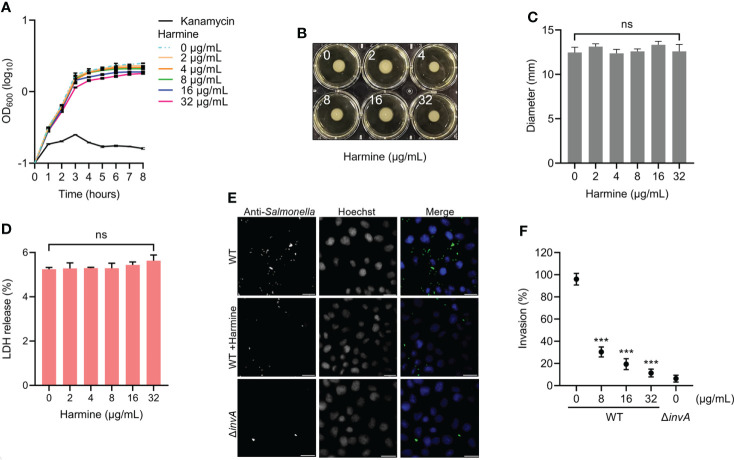
Harmine inhibits the invasion of SPI-1 T3SS-mediated *S.* Typhimurium into HeLa cells. **(A)** Effect of harmine on *S.* Typhimurium growth. Kanamycin (32 μg/mL) was used as a positive control. **(B)** Effect of harmine on *S.* Typhimurium motility. **(C)** Average the diameter of the colony is presented as the mean ± the standard deviation of three biological replicates. **(D)** Cytotoxicity of harmine on host cells was examined using LDH release assay. **(E)** Immunofluorescence analysis of the invasion of intracellular *S.* Typhimurium in HeLa cells with 32 µg/mL harmine (scale bar, 10 μm). **(F)** Plate counting method was used for analysis of the effect of harmine on bacterial invasion of HeLa cells. ***P < 0.001; ns, no significance.

Less than or equal to 32 μg/mL harmine did not affect the growth or mobility of SL1344 in LB broth (**Figures 5A-C**). The results of the LDH assay showed that 32 μg/mL harmine did not affect the viability of HeLa cells (**Figure 5D**). Therefore, we further investigated the mechanism of the concentration-dependent action of harmine on SPI-1. Taken together, the effect of harmine is independent of cytotoxicity toward HeLa cells and does not inhibit the normal growth of *S.* Typhimurium. Initial studies showed the ability of harmine to inhibit the expression of invasive proteins (**Figures 2**, **3**); thus, we further evaluated the invasion of HeLa cells because the invasion of cultured epithelial cells was predominantly related to SPI-1 T3SS effectors (Bueno et al., 2010). Next, we analyzed the inhibitory effects of harmine on the invasion of HeLa cells using a gentamicin protection assay (**Figures 5E, F**). The inhibition of intracellular SL1344 (green colonies) invasion was assessed by immunofluorescence analysis (**Figure 5E**). After preincubation of the bacteria with harmine, the number of harmine-pretreated WT-infected cells invading HeLa cells was significantly inhibited compared to that invading WT-infected cells (**Figure 5E**). Culturing with 8 μg/mL harmine significantly inhibited bacterial invasion compared to WT-infected bacteria, and a similar result was observed with the SL1344 Δ*invA* (**Figure 5F**). These results demonstrate that harmine can inhibit bacterial entry into host cells without affecting either bacterial viability or growth.

### Harmine alleviated *S.* Typhimurium-induced intestinal damage and improved the survival of infected mice

**Figure 6:**
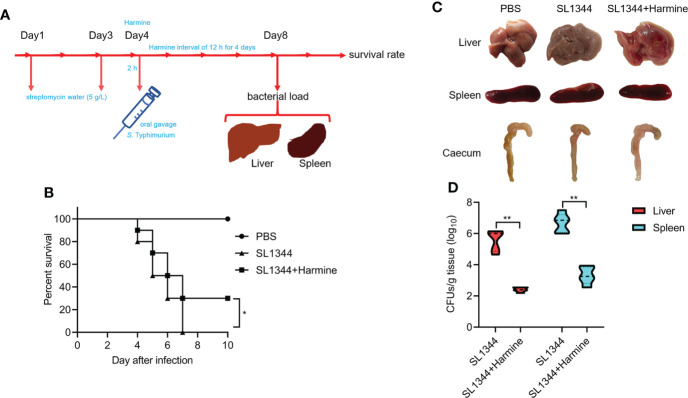
Harmine comprehensively protects mice from *S.* Typhimurium-mediated infection. **(A)** Illustration of the mouse infection assay. **(B)** Harmine prolonged the survival time and reduced the mortality of *S.* Typhimurium-infected mice (1×10^7^ CFUs, n=10 mice per group). **(C)** Autopsy analysis of tissues from *S.* Typhimurium-infected mice. **(D)** The bacterial load in the livers and spleens of infected mice (5×10^6^ CFUs, n=5 mice per group) was determined on the 4^th^ day after infection. *P < 0.05, **P < 0.01.

Harmine inhibited SL1344 invasion of HeLa cells, prompting us to evaluate whether harmine provided systemic protection. In a mouse infection model, the survival rate test was monitored for 10 days (**Figure 6A**). At Day 5 post infection, the mice infected with 1×10^7^ CFUs SL1344 had a 50% death rate, and the remaining SL1344 group mice did not survive past Day 7. In contrast, mice treated with harmine (100 mg/kg) for 4 consecutive days and the same dose of SL1344 presented a survival rate of 30% through Day 7 post infection. All mice survived in the blank control group (**Figure 6B**). Thus, these data clearly indicate that the oral administration of harmine improved the survival of mice subjected to the SL1344 infection *in vivo* model. Furthermore, the effect of harmine during SL1344 infection was observed on autopsy. Mice were sacrificed by cervical dislocation Day 4 after infection and macroscopic observations were performed to identify typical pathological features of the liver, caecum and spleen tissues of the mice (**Figure 6C**). The caecum from the PBS group was healthy and contained solidified feces. In contrast, the caecum of DMSO-treated mice after SL1344 infection presented less-solidified feces and atrophy, while less severe lesions were observed in the mice treated with harmine (**Figure 6C**). The liver remained healthy and ruddy in the harmine-treated mice after infection with SL1344. The livers of the SL1344 group were significantly blanched compared with those of the DMSO-treated group. These data demonstrate that the target organs presented less tissue damage. The bacterial load (liver and spleen) was reduced in the harmine-treated group compared with the infected group (**Figure 6D**). Collectively, these results indicated that harmine protects mice against infection with *S*. Typhimurium.

## Discussion

*Salmonella* can develop multidrug resistance, which has been a growing public health problem since the early 1990s (Koirala et al., 2012). The development of new treatment regimens is an alternative approach to addressing increases in bacterial infections and antibiotic resistance problems (Hu et al., 2017), especially those that only target pathogenic virulence factors without affecting bacterial survivability. Compared with antibiotics, most antivirulence agents do not affect bacterial growth (Rasko and Sperandio, 2010). A growing number of small molecular compounds targeting proteins in pathogenic bacteria T3SS are being screened from natural products (Duncan et al., 2012). For example, Myricanol at concentration of 100 μM inhibits the *S*. Typhimurium T3SS by interfering with the DNA-binding activity of HilD (Wu et al., 2020). Cytosporone B at concentration of 100 μM inhibits the transcription of SPI-1-related genes *via* the Hha–H-NS–HilD–HilC–RtsA–HilA regulatory pathway (Li et al., 2013). A 100 μM concentration of baicalein inhibits bacterial invasion of epithelial cells by targeting SPI-1 T3SS effectors and translocases (Tsou et al., 2016).

This study illustrated the mechanism of harmine against *S.* Typhimurium T3SS. Harmine was identified as an anti-*S.* Typhimurium T3SS inhibitor that does not affect bacterial growth. It inhibited the transcription and expression of HilA, consequently decreased the expression of the *S.* Typhimurium T3SS effector proteins SipA, SipB and SipC and ultimately inhibited the secretion of SPI-1 T3SS effectors and the invasion of *S.* Typhimurium at concentration of 150 μM. Harmine pretreatment significantly decreased the bacterial loads in mice infected with *S.* Typhimurium, improved the survival rate of *S.* Typhimurium-infected mice, and alleviated intestinal damage. Collectively, our results demonstrate that harmine inhibits T3SS function, reduces the pathogenicity of bacteria *in vitro* and provides mice with protection against a lethal dose of *S.* Typhimurium infection *in vivo*. Evidence on the therapeutic effect of harmine against *S.* Typhimurium infection has been provided, and it shows that harmine could represent a natural small molecule compound for the development of novel antibiotics targeting the T3SS. Harmine could be used in a variety of applications for the treatment of *Salmonella* infections. In the future, the pharmacokinetic parameters of harmine will also be elucidated by a mouse model. In addition, HilA, the transcriptional regulator of SPI-1, regulates the expression of SPI-1 apparatus and effector genes (Darwin and Miller, 1999; Eichelberg and Galán, 1999). Harmine can control the expression of SPI-1 apparatus genes, which inhibit the activity of the T3SS-1 machinery. Future studies should also focus on the mechanisms underlying the ability of harmine to target HilA and impair the virulence of *S.* Typhimurium.

In this study, harmine was proven for the first time to be an effective antivirulence and therapeutic drug for *S.* Typhimurium infection. Harmine acted as an anti-infection agent mainly by inhibiting the transcription and expression of the T3SS regulatory gene *hilA*, and it did not affect *S.* Typhimurium growth. Previous reports on the pharmacological effects of harmine have concentrated on its anxiolytic, behavioral, and antitumor effects.

We demonstrated for the first time that harmine is a novel *S.* Typhimurium T3SS inhibitor. The results demonstrate the molecular mechanism of this inhibitor on T3SS functions. The identification of the activity of harmine provides important insights on anti-T3SS virulence agents. Harmine has proven to be an encouraging candidate for new anti-T3SS drugs.

## Conclusions

Harmine inhibited T3SS functions by reducing the transcription and expression of the T3SS regulatory gene *hilA* and ultimately inhibited the invasion of *S.* Typhimurium *in vitro* and reduces tissue damage in *S.* Typhimurium-infected mice *in vivo*. Harmine could be a potential antivirulence drug for the development of treatments targeting *Salmonella* infection.

## Data availability statement

The original contributions presented in the study are included in the article/supplementary material. Further inquiries can be directed to the corresponding authors.

## Ethics statement

The animal study was reviewed and approved by The Institutional Animal Care and Use Committee of Jilin University (number of permit: 2021100893G).

## Author contributions

This study was conceived and designed by JQ and HL. The manuscript was written by YS and HL. The animal experiments were performed by YS, YL, JS and XC. The *in vitro* experiments were completed by YS and XC. The data were analyzed by HL, QL, and YS. The manuscript was revised by JQ, XD, YZ, and JW. All authors contributed to the article and approved the submitted version.

## Funding

This research was supported by the National Key Research & Development Program of China (2021YFD1801000), the Thousand Young Talents Program of the Chinese Government (JZQ) and startup fund from Jilin University.

## Conflict of interest

The authors declare that the research was conducted in the absence of any commercial or financial relationships that could be construed as a potential conflict of interest.

## Publisher’s note

All claims expressed in this article are solely those of the authors and do not necessarily represent those of their affiliated organizations, or those of the publisher, the editors and the reviewers. Any product that may be evaluated in this article, or claim that may be made by its manufacturer, is not guaranteed or endorsed by the publisher.

## References

[B1] Aiello D. Williams J. D. Majgier-Baranowska H. Patel I. Peet N. P. Huang J.. (2010). Discovery and characterization of inhibitors of *Pseudomonas aeruginosa* type III secretion. Antimicrob. Agents. Chemother. 54, 1988–1999. doi: 10.1128/AAC.01598-09 20176902 PMC2863679

[B2] Barthel M. Hapfelmeier S. Quintanilla-Martínez L. Kremer M. Rohde M. Hogardt M.. (2003). Pretreatment of mice with streptomycin provides a *Salmonella enterica* serovar typhimurium colitis model that allows analysis of both pathogen and host. Infect. Immun. 71, 2839–2858. doi: 10.1128/IAI.71.5.2839-2858.2003 12704158 PMC153285

[B3] Benzekri R. Bouslama L. Papetti A. Hammami M. Smaoui A. Limam F. (2017). Anti HSV-2 activity of *Peganum harmala* (L.) and isolation of the active compound. Microb. Pathog. 114, 291–298. doi: 10.1016/j.micpath.2017.12.017 29223449

[B4] Bueno S. M. Wozniak A. Leiva E. D. Riquelme S. A. Carreño L. J. Hardt W. D.. (2010). *Salmonella* pathogenicity island 1 differentially modulates bacterial entry to dendritic and non-phagocytic cells. Immunology. 130, 273–287. doi: 10.1111/j.1365-2567.2009.03233.x 20201987 PMC2878471

[B5] Chen D S. A. Fu Y. Wang X. Lv X. Xu W. Xu S.. (2015). Harmine blocks herpes simplex virus infection through downregulating cellular NF-κB and MAPK pathways induced by oxidative stress. Antiviral Res. 123, 27–38. doi: 10.1016/j.antiviral.2015.09.003 26348003

[B6] Coburn B. Grassl G. A. Finlay B. B. (2007). *Salmonella*, the host and disease: a brief review. Immunol. Cell. Biol. 85, 112–118. doi: 10.1038/sj.icb7100007 17146467

[B7] Darwin K. H. Miller V. L. (1999). Molecular basis of the interaction of *Salmonella* with the intestinal mucosa. Clin. Microbiol. Rev. 12 (3), 405–428. doi: 10.1128/CMR.12.3.405 10398673 PMC100246

[B8] Diepold A. Armitage J. P. (2015). Type III secretion systems: the bacterial flagellum and the injectisome. Philos.Trans. R. Soc Lond. B. Biol. Sci. 370 (1679), 20150020. doi: 10.1098/rstb.2015.0020 26370933 PMC4632597

[B9] Duncan M. C. Linington R. G. Auerbuch V. (2012). Chemical inhibitors of the type three secretion system: Disarming bacterial pathogens. Antimicrob. Agents. Chemother. 56, 5433–5441. doi: 10.1128/AAC.00975-12 22850518 PMC3486574

[B10] Eichelberg K. Galán J. E. (1999). Differential regulation of *Salmonella* typhimurium type III secreted proteins by pathogenicity island 1 (SPI-1)-encoded transcriptional activators InvF and HilA. Infect. Immun. 67 (8), 4099–4105. doi: 10.1128/IAI.67.8.4099-4105.1999 10417179 PMC96710

[B11] Ellermeier C. D. Ellermeier J. R. Slauch J. M. (2005). HilD, HilC and RtsA constitute a feed forward loop that controls expression of the SPI1 type three secretion system regulator hilA in *Salmonella enterica* serovar typhimurium. Mol. Microbiol. 57, 691–705. doi: 10.1111/j.1365-2958.2005.04737.x 16045614

[B12] Ellermeier C. D. Slauch J. M. (2003). RtsA and RtsB coordinately regulate expression of the invasion and flagellar genes in *Salmonella enterica* serovar typhimurium. J. Bacteriol 185, 5096–5108. doi: 10.1128/JB.185.17.5096-5108.2003 12923082 PMC181000

[B13] Ellermeier J. R. Slauch J. M. (2007). Adaptation to the host environment: regulation of the SPI1 type III secretion system in *Salmonella enterica* serovar typhimurium. Curr. Opin. Microbiol. 10, 24–29. doi: 10.1016/j.mib.2006.12.002 17208038

[B14] Filali I. Bouajila J. Znati M. Bousejra-El Garah F. Ben Jannet H. (2015). Synthesis of new isoxazoline derivatives from harmine and evaluation of their anti-Alzheimer, anti-cancer and anti-inflammatory activities. J. Enzyme. Inhib. Med. Chem. 30, 371–376. doi: 10.3109/14756366.2014.940932 25068731

[B15] Galan J. E. Curtiss R. (1989). Cloning and molecular characterization of genes whose products allow *Salmonella* typhimurium to penetrate tissue culture cells. Proc. Natl. Acad. Sci. 86, 6383–6387. doi: 10.1073/pnas.86.16.6383 2548211 PMC297844

[B16] Golubeva Y. A. Sadik A. Y. Ellermeier J. R. Slauch J. M. (2012). Integrating global regulatory input into the *Salmonella* pathogenicity island 1 type III secretion system. Genetics. 190, 79–90. doi: 10.1534/genetics.111.132779 22021388 PMC3249375

[B17] Hamsa T. P. Kuttan G. (2011). Harmine activates intrinsic and extrinsic pathways of apoptosis in B16F-10 melanoma. Chin. Med. 6, 11. doi: 10.1186/1749-8546-6-11 21429205 PMC3076298

[B18] Hu Y. Huang H. Cheng X. Shu X. White A. P. Stavrinides J.. (2017). A global survey of bacterial type III secretion systems and their effectors. Environ. Microbiol. 19, 3879–3895. doi: 10.1111/1462-2920.13755 28401683

[B19] Kim K. Golubeva Y. A. Vanderpool C. K. Slauch J. M. (2019). Oxygen-dependent regulation of SPI1 type three secretion system by small RNAs in *Salmonella enterica* serovar typhimurium. Mol. Microbiol. 111, 570–587. doi: 10.1111/mmi.14174 30484918 PMC6417950

[B20] Koirala K. D. Thanh D. P. Thapa S. D. Arjyal A. Karkey A. Dongol S.. (2012). Highly resistant *Salmonella enterica* serovar typhi with a novel gyrA mutation raises questions about the long-term efficacy of older fluoroquinolones for treating typhoid fever. Antimicrob. Agents. Chemother. 56, 2761–2762. doi: 10.1128/AAC.06414-11 22371897 PMC3346606

[B21] Li J. Lv C. Sun W. Li Z. Han X. Li Y.. (2013). Cytosporone b, an inhibitor of the type III secretion system of salmonella enterica serovar typhimurium. Antimicrob. Agents. Chemother. 57, 2191–2198. doi: 10.1128/AAC.02421-12 23459474 PMC3632957

[B22] Ma Y. Wink M. (2010). The beta-carboline alkaloid harmine inhibits BCRP and can reverse resistance to the anticancer drugs mitoxantrone and camptothecin in breast cancer cells. Phytother. Res. 24, 146–149. doi: 10.1002/ptr.2860 19548284

[B23] Moest T. P. Méresse S. (2013). Salmonella T3SSs: successful mission of the secret(ion) agents. Curr. Opin. Microbiol. 16, 38–44. doi: 10.1016/j.mib.2012.11.006 23295139

[B24] Negrea A. Bjur E. Ygberg S. E. Elofsson M. Wolf-Watz H. Rhen M. (2007). Salicylidene acylhydrazides that affect type III protein secretion in *Salmonella enterica* serovar typhimurium. Antimicrob. Agents. Chemother. 51, 2867–2876. doi: 10.1128/AAC.00223-07 17548496 PMC1932493

[B25] Nesterenko L. N. Zigangirova N. A. Zayakin E. S. Luyksaar S. I. Gintsburg A. L. (2016). A small-molecule compound belonging to a class of 2,4-disubstituted 1,3,4-thiadiazine-5-ones suppresses *Salmonella* infection *in vivo* . J. Antibiot 69, 422–427. doi: 10.1038/ja.2015.131 26732253

[B26] Plym F. L. Wierup M. (2006). *Salmonella* contamination: A significant challenge to the global marketing of animal food products. Rev. Sci. Tech 25, 541–554. doi: 10.20506/rst.25.2.1683 17094696

[B27] Quintana V. M. Piccini L. E. Zénere J. P. Damonte E. B. Ponce M. A. Castilla V. (2016). Antiviral activity of natural and synthetic β-carbolines against dengue virus. Antiviral. Res. 134, 26–33. doi: 10.1016/j.antiviral.2016.08.018 27568370

[B28] Rasko D. A. Sperandio V. (2010). Anti-virulence strategies to combat bacteria-mediated disease. Nat. Rev. Drug Dis. 9, 117–128. doi: 10.1038/nrd301 20081869

[B29] Réus G. Z. Stringari R. B. de Souza B. Petronilho F. Dal-Pizzol F. Hallak J. E.. (2010). Harmine and imipramine promote antioxidant activities in prefrontal cortex and hippocampus. Oxid. Med. Cell. Longev 3, 325–331. doi: 10.4161/oxim.3.5.13109 21150338 PMC3154037

[B30] Riquelme S. Varas M. Valenzuela C. Velozo P. Chahin N. Aguilera P.. (2016). Relevant genes linked to virulence are required for *Salmonella* typhimurium to survive intracellularly in the social amoeba dictyostelium discoideum. Front. Microbiol. 7. doi: 10.3389/fmicb.2016.01305 PMC4993766 27602025

[B31] Schechter L. M. Lee C. A. (2001). AraC/XylS family members, HilC and HilD, directly bind and derepress the *Salmonella* typhimurium hilA promoter. Mol. Microbiol. 40, 1289–1299. doi: 10.1046/j.1365-2958.2001.02462.x 11442828

[B32] Sockett P. N. (1995). The epidemiology and costs of diseases of public health significance, in relation to meat and meat products. J. Food. Saf. 15, 91–112. doi: 10.1111/j.1745-4565.1995.tb00126.x

[B33] Tsou L. K. Lara-Tejero M. RoseFigura J. Zhang Z. J. Wang Y. C. Yount J. S.. (2016). Antibacterial flavonoids from medicinal plants covalently inactivate type III protein secretion substrates. *J. Am. Chem* . Soc. 138 (7), 2209–2218. doi: 10.1021/jacs.5b11575 PMC4831573 26847396

[B34] Walther B. Tedin K. Lübke-Becker A. (2016). Multidrug-resistant opportunistic pathogens challenging veterinary infection control. Vet. Microbiol. 200, 71–78. doi: 10.1016/j.vetmic.2016.05.017 27291944

[B35] Wu Y. Yang X. Zhang D. Lu C. (2020). Myricanol inhibits the type III secretion system of *Salmonella enterica* serovar typhimurium by interfering with the DNA-binding activity of HilD. Front. Microbiol. 11. doi: 10.3389/fmicb.2020.571217 PMC7546796 33101243

[B36] Yahr T. L. Wolfgang M. C. (2006). Transcriptional regulation of the *Pseudomonas aeruginosa* type III secretion system. Mol. Microbiol. 62, 631–640. doi: 10.1111/j.1365-2958.2006.05412.x 16995895

[B37] Zaidi M. I. Gul A. Ali S. (2004). Antifungal activity of harmine, HgCl2 and their complex. Sarhad. J. Agric. 20 (4), 623–626.

